# Spray cooling characteristics of nanofluids for electronic power devices

**DOI:** 10.1186/s11671-015-0793-7

**Published:** 2015-03-19

**Authors:** Shou-Shing Hsieh, Hsin-Yuan Leu, Hao-Hsiang Liu

**Affiliations:** Department of Mechanical and Electromechanical Engineering, National Sun Yat-Sen University, Kaohsiung, 80424 Taiwan

**Keywords:** Spray heat transfer, Ag/MCNT nanofluids, Transient boiling, Cooling enhancement

## Abstract

The performance of a single spray for electronic power devices using deionized (DI) water and pure silver (Ag) particles as well as multi-walled carbon nanotube (MCNT) particles, respectively, is studied herein. The tests are performed with a flat horizontal heated surface using a nozzle diameter of 0.5 mm with a definite nozzle-to-target surface distance of 25 mm. The effects of nanoparticle volume fraction and mass flow rate of the liquid on the surface heat flux, including critical heat flux (CHF), are explored. Both steady state and transient data are collected for the two-phase heat transfer coefficient, boiling curve/ cooling history, and the corresponding CHF. The heat transfer removal rate can reach up to 274 W/cm^2^ with the corresponding CHF enhancement ratio of 2.4 for the Ag/water nanofluids present at a volume fraction of 0.0075% with a low mass flux of 11.9 × 10^−4^ kg/cm^2^s.

## Background

Spray cooling is an efficient way to remove high heat flux from heated surfaces. Frequently, the essential requirements for many electronic power devices are a small surface superheat and a low mass flow rate. It has long been recognized [1] that spray cooling with phase change has been demonstrated to be a powerful method to remove high heat flux from modified surfaces, using water as a coolant with a higher mass flux.

Three different regimes have been termed in boiling spray cooling: nucleate boiling from surface and secondary sites, convection heat transfer, and direct evaporation from the liquid film over the surface [2]. Many studies were conducted on the influence of the spray parameters on the cooling heat flux. It was found that the volumetric spray flux has a major effect on the heat transfer [2,3] compared to those of hydrodynamic parameters of spray [1]. Still, investigators believe that spray cooling performance and critical heat flux (CHF) usually depend on a number of parameters, including the following: nozzle type, nozzle-to-surface distance, heated surface condition, working liquid, and droplet dynamics [4,5]. Applications exist in a wide range of industrial processes, including rapid cooling and quenching in metal foundries, emergency core cooling systems, cooling of microelectronics, and the ice chiller in air-conditioning systems.

The physical process of spray cooling, due to the impact of in-flight droplet impingings onto a heated surface, consequently may lead to splashing, spreading, or rebounding [6]. Obviously, the rebound process would result in decreased liquid cooling capacity and efficiency. The impinging droplets spread on the surface and can form a continuous liquid film. At high wall superheat, a thin vapor layer can form under the droplets or the thin liquid films due to boiling [7].

Advances in nanofabrication processes have led to many innovations in spray and atomization technologies. Nanofluids are fluids that contain nanoparticles, such as metals, oxides, carbides, and nitrides, with sizes less than 100 nm. They are known to have higher thermal conductivity compared to that of the base fluid; hence, the enhancement of their thermal conductivity at room temperature was considered in the majority of the research [8]. In addition, the application of nanofluids in spray cooling for electronic devices is an emerging area of research [9]. In fact, some metals and non-metals, like gold, silver, copper, aluminum, and carbon, have been found to have quite high thermal conductivity compared to cooling liquids like water, engine oil, and ethylene. Therefore, small amounts of these materials with high thermal conductivity added to base fluids like water would increase the thermal conductivity of the base fluids without the problems encountered in common slurries, such as clogging, erosion, sedimentation, and a large increase in pressure drop.

As stated previously, the addition of metal/or metal oxide nanoparticles to a liquid coolant is one of the notable examples proffered to increase the mixture’s thermal conductivity and possibly increase the heat transfer. Although several investigators [10,11] have proven this concept, quite a few results show an opposite trend [12-14] due to nanoparticle deposition on the surface impeding heat transfer performance. In addition, inconsistency in the heat transfer performance by nanofluids with spray cooling can also be found [9,15]. Based on the findings above, it may be concluded that the heat transfer coefficient increase/or decrease from the addition of nanoparticles depends on either the base fluid used or the target surface temperatures and the spray duration time on the surface**/**or the nanofluid impact velocity. Although the results are inconsistent with respect to boiling enhancement, both results may be true in their respective particle concentration range, because these two ranges may be dominated by different phenomena which result in different heat transfer characteristics. Moreover, it has been shown [4] that the CHF is enhanced for the pool boiling because the deposition of nanoparticles on the heated surface results in a change in the surface properties including capillarity and coatability. The contact angle, therefore, decreases for a nucleate boiling in nanofluids.

Although there are plenty of advantages of spray cooling over existing cooling techniques, it appears that there is a very limited knowledge base with contrary experimental data on spray impingement cooling of surfaces for situations when the coolant of nanoparticle and liquid mixtures has a very low volume concentration (0.0075%) of nanopowder, especially for metal (like Ag) and MCNT nanoparticles. In fact, Ag/water nanofluid spray has not been seen in publications. In view of the foregoing discussion, this paper presents a relatively detailed study on the spray impingement heat transfer, both steady and transient, to broaden our fundamental understanding of the two-phase spray cooling of nanofluids. In order to accomplish this goal, experiments were performed with Ag/MCNT nanoparticles and a deionized (DI) water mixture, respectively, with different particle volume fractions. Furthermore, the influence of the liquid mass flux on the heat transfer performance was examined. In the following section, the experimental setup is described; in Section ‘Results and discussion,’ experimental results are presented and discussed.

## Methods

The preparation of Ag/MCNT nanoparticles and deionized water mixtures, as well as the experimental setup and procedures, is presented in this section.

### Silver/DI water and MCNT/DI water nanofluids

**Figure 1:**
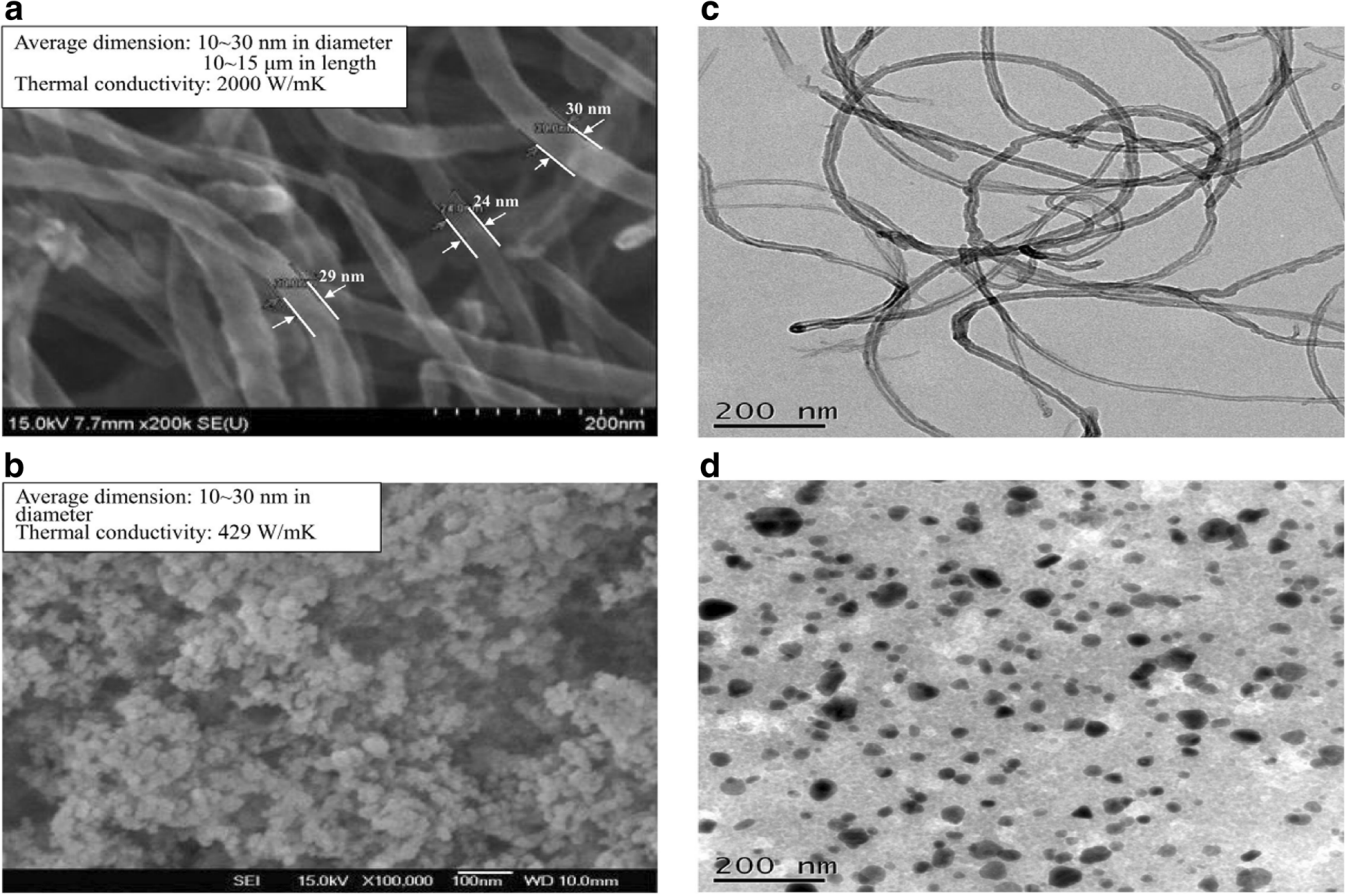
**SEM (before)/TEM (after) image. (a)** MCNT nanopowder, **(b)** Ag nanopowder, **(c)** MCNT nanofluids, **(d)** Ag nanofluids.

Ag/MCNT nanoparticles are very chemically stable (see Table 1 for details) and can be easily dispersed in DI water (base fluid) to form colloidal nanofluids. The properties of the base fluid (DI water) used are listed in Table 2. In the experiments, set amounts of Ag/MCNT nanoparticles were added to the base fluid, deionized (DI) water, to form three volume fractions (*ϕ*) of 0.0025, 0.005, and 0.0075 vol.%, respectively, which were ultrasonically excited for 24 h to ensure proper homogenization of the nanoparticles to obtain a stable and uniform colloidal solution with an ultrasonic vibrator (D9NX-DC200H, DELTA NEW INSTRUMENT CO., Ltd, New Taipei City, Taiwan). Note that no surfactant was used in the experiments. The nanoparticles used were manufactured by Vanung University, Taiwan. *ϕ* can be expressed as *ϕ* = volume of Ag/or CNT nanoparticles/(volume of Ag/or MCNT nanoparticles + volume of DI water). The properties of the nanofluids are presented in Table 3. Scanning electron microscope (SEM)/transmission electron microscope (TEM) examinations of the nanofluids for characterization used in this study are shown in Figure 1a,b for MCNT and silver powder and Figure 1c,d for MCNT nanofluids and Ag nanofluids, respectively, before/after becoming colloidal solutions. The applied voltage was 15 kV with a magnification of × 200,000 and × 100,000 for MCNT and Ag nanopowder, respectively, for the (SEM) (HITACHI S-3000H, Tokyo, Japan); while, for the (TEM) (PHILIPS CM-200 TWIN TEM, Shanghai, People’s Republic of China), an applied voltage of 200 kV with × 150,000 magnification was used for MCNT and Ag nanofluids, respectively. During nanofluid solution preparation, the colloidal solution temperature was maintained at a room temperature of 25°C. Obviously, the Ag nanofluid solution would be more uniform, which indicates that better mixing and an evenly dispersed phase without agglomeration was obtained (see Figure 1c,d for details). The nanoparticles that were stuck to the heated surface were removed before running subsequent experiments.

**Table 1:** **Relevant parameters of the nanopowder used (at 25°C, 1 atm)**

	**Ag nanoparticles**	**Multi-walled carbon nanotube (MCNT) particles**
Average dimension in water	10 ~ 30 (nm)	10 ~ 30 (nm) in diameter
		10 ~ 15 (μm) in length
Surface ratio (m^2^/g)	≥150	≥200
Density (g/cm^3^)	10.49	2.6
Melting point (°C)	961	3,550
Specific heat capacity (kJ/kgK)	0.235	0.45
Thermal conductivity (W/mK)	429	2,000

**Table 2:** **Thermodynamic properties of the base fluid (DI water at 25°C and 1 atm)**

**Properties**	**DI water**
Average molecular weight (kg/kg mol)	18.16
Critical temperature (°C)	374.2
Saturation temperature (°C)	99.9
Density of liquid (kg/m^3^)	997
Heat of vaporization (kJ/kg)	2256.7
Thermal conductivity of liquid (W/mK)	0.606
Specific heat of liquid (kJ/kgK)	4.22
Thermal diffusivity of liquid (m^2^/s)	1.440 × 10^−7^
Surface tension of liquid (N/m)	0.072
Viscosity (Ns/m^2^)	8.9 × 10^−4^

**Table 3:** **Properties of the nanofluid (at 25°C, 1 atm)**

	**Ag**			**MCNT**		
Volume fraction, *ϕ* (vol.%)	0.0025	0.0050	0.0075	0.0025	0.0050	0.0075
Density, *ρ*_nf_ (kg/m^3^)	997.24	997.47	997.71	997.04	997.08	997.12
Heat capacity, *C*_p,nf_ (kJ/kg-K)	4.19990	4.19980	4.19970	4.19991	4.19981	4.19972
Viscosity, *μ*_nf_ (N-s/m^2^)	8.90056 × 10^−4^	8.90111 × 10^−4^	8.90167 × 10^−4^	8.90028 × 10^−4^	8.900056 × 10^−4^	8.90083 × 10^−4^
Thermal conductivity, *k*_nf_ (W/m-K)	0.6060	0.6061	0.6062	0.6078	0.6095	0.6113
Prandtl number, Pr_nf_	6.16810	6.16788	6.16766	6.15041	6.13260	6.11490
Surface tension, *σ*(N/m) (measured)	0.129	0.097	0.089	0.078	0.068	0.063
Surface area, (m^2^/100 g solution)	3.9	7.8	11.7	1.3	2.6	3.9
Density, *ρ*_nf_	*ρ*_nf_ = (1 − φ)*ρ*_bf_ + φ*ρ*_np_ [16]					
Heat capacity, *C*_p,nf_	*C*_p. nf_ = (1 − φ)*C*_p,bf_ + φ*C*_p,np_ [17]					
Viscosity, *μ*_nf_	*μ*_nf_ = *μ*_bf_(1 + 1.25 φ) for MCNT [18]					
	*μ*_nf_ = *μ*_bf_(1 + 2.5 φ) for Ag [19]					
Thermal conductivity, *k*_nf_	${k}_{\mathrm{nf}}={k}_{\mathrm{bf}}\frac{k_{\mathrm{np}}/{k}_{\mathrm{bf}}+K-K\upvarphi \left(1-{k}_{\mathrm{np}}/{k}_{\mathrm{bf}}\right)}{k_{\mathrm{np}}/{k}_{\mathrm{bf}}+K+\upvarphi \left(1-{k}_{\mathrm{np}}/{k}_{\mathrm{bf}}\right)}$ where $K=2\ {\upvarphi}^{0.2}\left(\frac{1}{d}\right)$ for MCNT [20] ${k}_{\mathrm{nf}}={k}_{\mathrm{bf}}\frac{k_{\mathrm{np}}+2{k}_{\mathrm{bf}}-2\upvarphi \left({k}_{\mathrm{bf}}-{k}_{\mathrm{np}}\right)}{k_{\mathrm{np}}+2{k}_{\mathrm{bf}}+\upvarphi \Big({k}_{\mathrm{bf}}-{k}_{\mathrm{np}\Big)}}$ for Ag [21]					

nf, nanofluid; bf, base fluid; np, nanoparticle; l, the length of MCNT; d, the diameter of MCNT.

### Experimental setup

**Figure 2:**
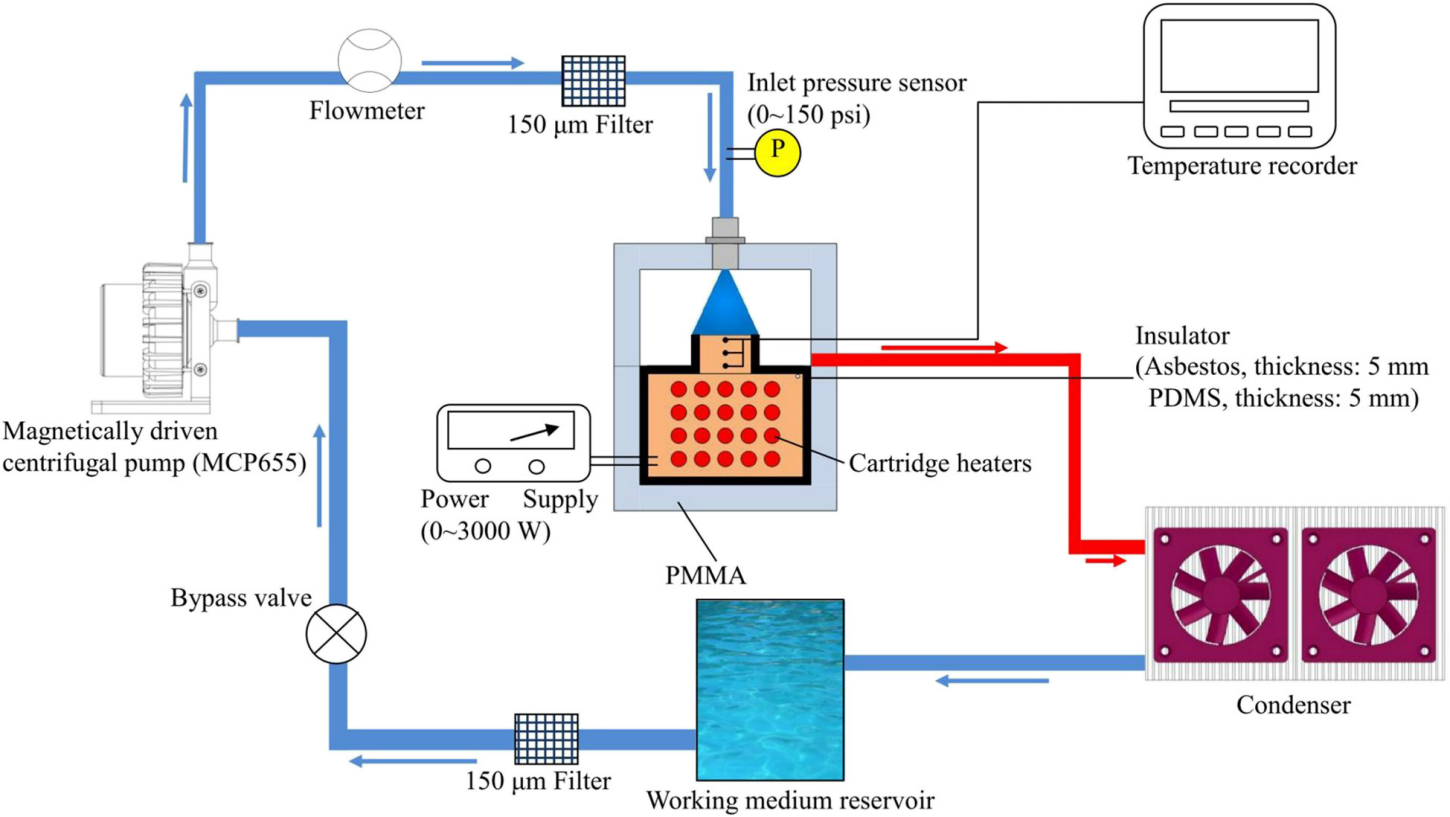
**Schematic of the experimental setup and flow loop.**

A schematic of the experimental setup used is shown in Figure 2. As illustrated, the system comprised a nanofluid flow loop and a test spray chamber including: a heating block, a cooling water loop, and a data acquisition system. The nanofluid flow loop was a closed circulation and was so designed that the liquid entered the test section at the desired flow rate and temperature. In order to guarantee that the concentration of nanofluid was still constant during experiments, special care was taken through sampling measurement after experiments for each case.

**Figure 3:**
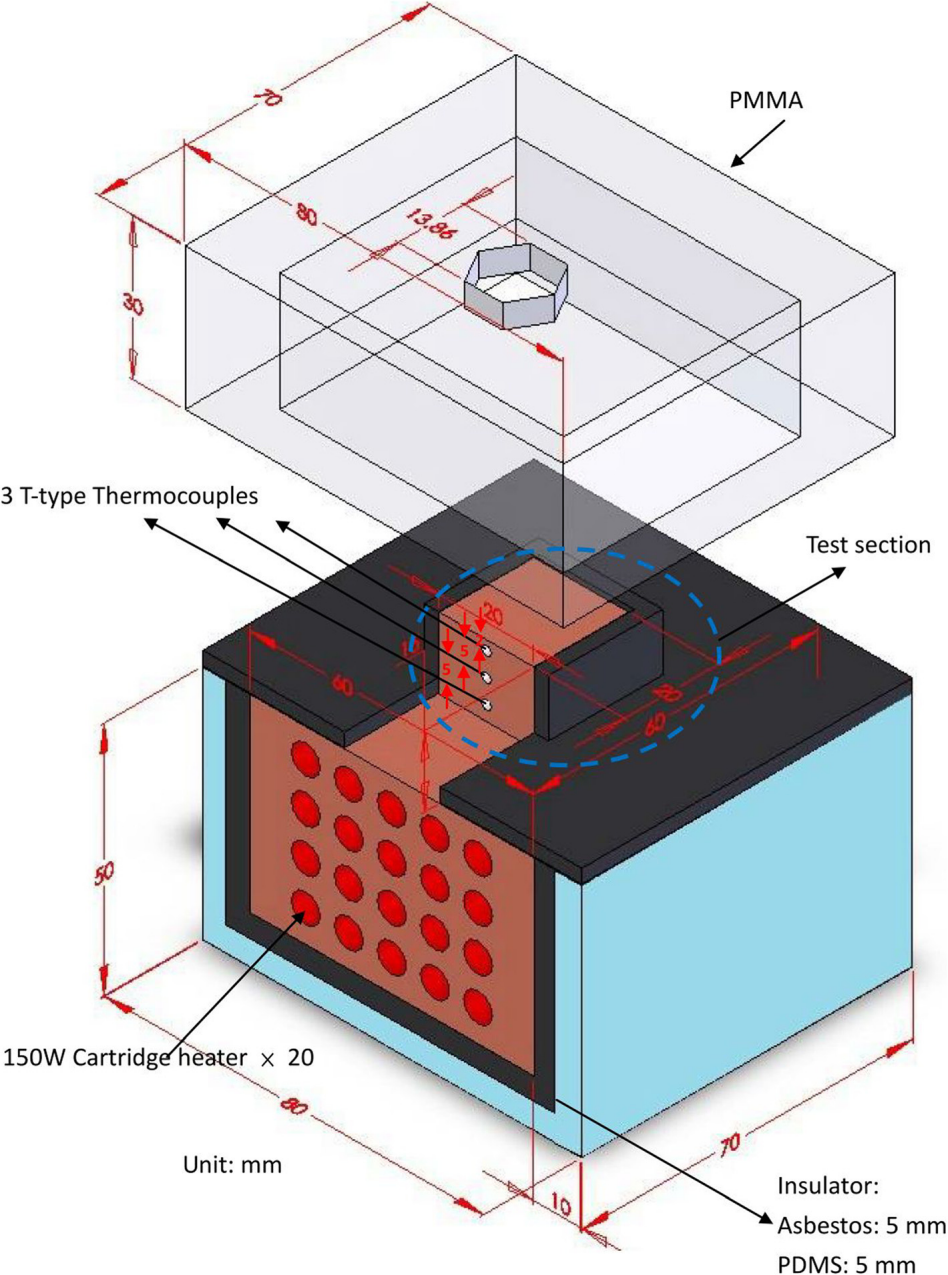
**Heater details.**

As shown in Figure 3, 20 cartridge heaters (150 W each), manufactured by SHINNCHYUAN ELECTRIC WORKS Ltd., were inserted evenly into the insulated copper block to generate 3 kW of uniform load. Except for the heated surface, the entire copper block was wrapped with a fiberglass insulating blanket to prevent heat loss from the heater. The upper end face in contact with the spray had the dimensions of 1.0 × 1.0 cm^2^ for its test surface area. The dissipated heat flux and surface temperature were estimated using three T-type 80 μm (OMEGA Engineering Inc., model no. TT-T-40, Stamford, USA) thermocouples spaced 5 mm apart along the axis of the copper block. The upper thermocouple was positioned 2 mm beneath the heater surface. These three T-type thermocouples were inserted into 1.8-mm diameter holes drilled on the reduced section of the copper block. The holes were filled with a resin (PK-3 thermal compound, Prolimatech, Taipei, Taiwan) with a high thermal conductivity (approximately 11 W/mK) to avoid air gaps inside the holes. Moreover, the measured temperature distributions in the copper block with three 1.8-mm diameter holes filled with PK-3 thermal compound fillers were compared with a copper block without holes to examine the effect of the thermocouples/holes with fillers on the temperature field. Results showed that the hole effect can be negligible. The surface temperature was justified via a commercial 3D computer code, and it was found that the space variation was quite small. Based on 1D heat conduction, the dissipated heat flux was estimated based on the three temperature measurements and their corresponding distances. Similarly, the surface temperature was extrapolated based on the above stated three measured temperatures with an error of ±8.4%.

Spray tests were performed using a full cone spray circular nozzle (JX005, Ikeuchi Taiwan Co. Ltd. Taipei, Taiwan) with a diameter of 0.5 mm and a fixed cone angle of 50° to achieve the desired mass flux sprayed onto the test surface. A nozzle-to-surface distance of 25 mm was tested. Two different geometrical shapes of nanofluids were used. One was spherical (Ag) and the other cylindrical (MCNT); the results were as follows: one was DI water with Ag particles with an average diameter of 15 nm and the other was DI water with CNT particles with an average diameter of 10 nm and an average length of 250 nm. Three different particle volume fractions of 0.0025, 0.005, and 0.0075 vol.% were examined. Care was taken to properly polish the heated surface before/after the experiment with emery paper (mesh number 300), and then it was cleaned with acetone. A flow meter was applied to the delivery side to control the fluid flow rate and pressure of the spray nozzle. The test section was placed into a PMMA tank, spray chamber, which was equipped with a pressure gauge, safety valve, and a relief drainage valve. Experiments involved a high subcooling system (the temperature of nanofluids at nozzle exit was fixed at 25°C). The spray chamber was maintained at atmospheric pressure throughout the study.

### Spray parameter

The average velocity of spray droplets impinged on the test surface was estimated by the following equation [22]. The spray mass flux at the test surface was calculated for different pressure drops (Δ*P*) by replacing the test surface with a section of copper tubing whose internal diameter was the same as the diameter of the surface; as well, the tubing pressure was identical to the chamber pressure, and the volume of liquid flowing into the tubing with a known time period was recorded. Repeatable tests were made to warrant its accuracy. The maximum uncertainty above was estimated to be less than ±5% including vapor escape at high heat fluxes.

Three statistical mean diameters of the spray droplets are frequently encountered: the number mean number *d*_10_; the volume mean diameter *d*_30_; and the Sauter (or surface weighted) mean diameter (SMD), *d*_32_. *d*_32_ was used to calculate the spray Weber number. It was provided by the nozzle supplier (JJXP 005, Ikeuchi Taiwan Co., Ltd.), and the Weber number was defined, *ρ*_l_*u*_0_^2^*d*_32_/*σ*. The SMD (*d*_32_) was selected to represent the mean diameter of droplets within the cell and is quite relevant to hydrodynamics and mass transfer due to both drag and reaction rates being proportional to the spray droplet area. The spray parameters mentioned above, with other relevant variables, are listed in Table 4. Strictly speaking, the present Weber number is temperature and nanofluid dependent, but, for simplicity, it was assumed to be constant and the property used was based on the base fluid (i.e. DI water), although the surface tension of the nanofluid at different volume concentrations had been measured in the present study.

**Table 4:** **Related variables and parameters used in the study**

**Experimental parameters**			
Heat spreader area (cm^2^)	4		
Nozzle to target surface distance (mm)	25		
Nozzle diameter, *dj* (μm)	500		
Working medium	DI water		
Pressure difference, Δ*P* (kPa)	18.6	32.4	53.1
Volumetric flow rate, *Q* (ml/s)	2.63	3.57	4.76
Mass flux, *G* (kg/cm^2^s)	6.58 × 10^−4^	8.93 × 10^−4^	11.90 × 10^−4^
Droplet diameter, *d*_32_ (μm)	299.8	259.7	228.5
Weber number^a^, We = *ρu*_m_^2^ *d*_32_/*σ*	140.2	223.9	350.3
Ohnesorge number^a^, Oh = We^0.5^/Re	0.0038	0.0036	0.0034
Reynolds number^a^, Re = *ρu*_0_ *d*_j_/μ	7784.7	10572.8	14097.1
Saturation temperature, *T*_sat_ (°C)	100		
Saturation pressure, *P*_sat_ (kPa)	101		
Degree of subcooling, Δ*T*_sub_ (°C)	75		
Characterization of the heated surface			
Material	Cu		
Thermal conductivity (W/mK)	401		
Thermal expansion coefficient (×10^−6^/K)	17		
Specific heat capacitance (J/gK)	0.385		
Density (kg/m^3^)	8,920		
Melting point (°C)	1,084		
Roughness (μm)	2 ~ 3		

^a^Only for base water(can also be calculated for nanofluids).

### Experimental procedure

During spray cooling, the copper target surface was first heated to approximately 300°C by the cartridge heaters; and then, nanofluid from the spray nozzle was sprayed on the testing surface; only the surface of the open area was cooled down by the nanofluid coolant. The fluid was collected after spraying and recycled back to the tank. The temperature measurement was stopped after the testing surface was cooled down to 25°C. It usually takes a few hundreds of seconds. The range of temperature measured by T type was 0 to 700 K. A data logger (INTELLILOGGER IL-80, LOGIC BEACH, Inc. La Mesa, USA) recorded from three thermocouples at 100-ms intervals. The repeatability of the uncertainty is within ±0.1°C. Both the steady-state boiling curve and transient cooling curves were measured. To obtain a steady state measurement, all temperature measurements were taken at least 300 s after the onset of spraying or any change in the heat impact. A series of measurements was performed while changing the mass flux.

For transient, as stated previously, the heated surface was heated to 300°C by first regulating the power to the heaters. Then, the water supply pump was switched on. Once the nozzle pressure reached a steady value, the power to the heaters was turned off allowing water to spray from the nozzle to simultaneously quench the heated surface. It usually takes 300 s, depending on spray parameters, to cool the heated surface from 300°C to 60°C. Temperatures measured by thermocouples were continuously recorded by a data acquisition system.

### Data reduction and uncertainty analysis

The heat transfer rate can be calculated at the heated surface [23] based on Fourier’s law:

1 $$ {k}_{\mathrm{cu}}{\left(\frac{dT}{dx}\right)}_{x={0}^{+}}={q}^{{\prime\prime} } $$

Here, *k*_cu_ is the thermal conductivity of the copper plate, *Tw* is the heated surface temperature, and *Tc* is the average temperature of the spray liquid layer on the heated surface. The temperature gradients, (dT/dx)_*x* = 0+_ at the surface temperature of the copper plate, *Tw*, were determined by extrapolation of the best fit through temperature measurements acquired simultaneously along the thickness of the plate with an uncertainty of ±1.2%. Equation 1 indicates the heat flux conducted through the copper plate and through heat transfer coefficient *h*, due to the convective and evaporative heat transfer mechanisms. For transient cooling, results from Equation 1 were rechecked for the worst case via a 2D inverse heat conduction (IHC) technique implemented with finite difference methods [8] by using the measured temperature as the input to a finite difference IHC model to inversely calculate surface heat flux/surface temperature. It was found that the deviation of the surface temperature was less than 3%.

Average heat transfer coefficient for the spray cooling was determined for each experimental run from the following relation:

2 $$ \overline{h}={q}_s^{{\prime\prime} }/\left({T}_w-{T}_{\mathrm{sat}}\right) $$

where the average surface heat flux *q*″, obtained from Equation 1, and the heated surface temperature were determined from the parabolic curve fit of the centerline temperature; *T*_sat_ is the spray saturation temperature. The present heat leak was found to be negligible.

The major uncertainties in this study were due to uncertainties in water flow rate, heated surface temperature, and heat flux measurements. The water flow rate was steady to within ±2% over the range of flow rates tested. The error in temperature difference between the spray and the heated surface was estimated to be less than 3%. The total uncertainty for the heat flux was estimated to be less than ±5.2%. Detailed uncertainty of relevant parameters/variables is listed in Table 5.

**Table 5:** **Measurement uncertainty for relevant parameters/variables**

**Parameters/variables**		**System error**	**Random error**	**Combined error**
Test surface	Length (*L*)	±0.05%	±0.94%	±0.94%
	Width (*W*)	±0.05%	±0.88%	±0.88%
	High (*H*)	±0.5%	±4.58%	±4.61%
	Area (*A*)	±0.1%	±1.82%	±1.82%
Test chamber	Length (*L*)	±0.02%	±1.05%	±1.05%
	Width (*W*)	±0.02%	±1.27%	±1.27%
	High (*H*)	±0.04%	±3.20%	±3.2%
	Distance between thermocouples (Δ*x*)	±0.2%	±1.17%	±1.19%
Measurement parameters and variables	Surface temperature (*Tw*)	±3.87%	±7.40%	±8.35%
	Power (*P*)	±1.91%	±3.02%	±3.57%
	Heat flux (*q*″)	±2.01%	±4.84%	±5.24%
	Volumetric flow rate (*Q*)	±1.60%	±2.74%	±3.17%
	Mass flux (*G*)	±1.70%	±4.56%	±4.87%
	Pressure drop (Δ*P*)	±0.18%	±1.42%	±1.43%
Dimensionless group/heat transfer data	Weber number (We)	±1.33%	±2.58%	±2.9%
	Reynolds number (Re)	±1.72%	±5.03%	±5.32%
	Heat transfer coefficient (*h*)	±5.88%	±12.24%	±13.58%

## Results and discussion

### Cooling curves (transient)

#### Base fluid (three different mass fluxes)

**Figure 4:**
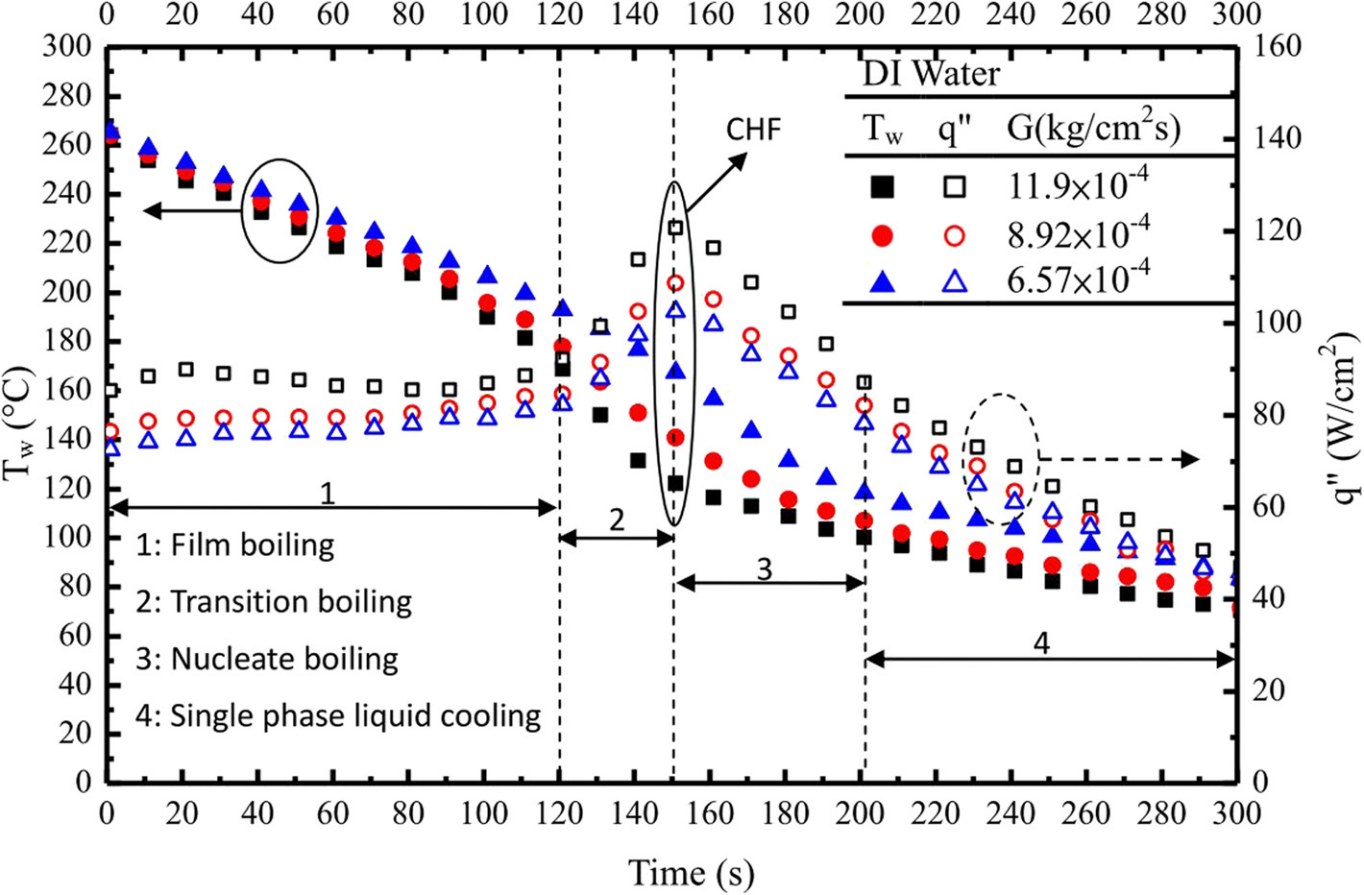
**Base fluid (DI water) cooling curve (boiling curves included).**

For DI water, low mass flux varying from 6.57 and 11.90 × 10^−4^ kg/cm^2^s and temperature differences for 60°C to 280°C, the cooling curves were measured. These data were used as reference data to assess the heat transfer/cooling performance of nanofluids. Typical results, shown in Figure 4, represent the cooling history of the surface temperature with DI water (numerals indicate the cooling regime). Also included in Figure 4 are the corresponding boiling curves. For Figure 4, the cooling rate in the stable film boiling regime increases with the increase in working fluid mass flux. The onset of the transition boiling was identified by the change in the cooling rate (slope change) at 180°C (*G* = 8.92 × 10^−4^ kg/cm^2^s); at this temperature, it takes about 120 s for DI water from the start. As the surface temperature decreases from Leidenfrost in the transition boiling regime, the cooling rate increases as more efficient surface wetting and boiling occur. At the lower temperature boundary of the transition boiling regime, where the entire surface becomes occupied by wetting, the cooling rate reaches a maximum (so called CHF). This maximum heat flux can be seen from the steepest portion of the cooling curves in Figure 4. Below CHF (e.g. *G* = 8.92 × 10^−4^ kg/cm^2^s), the cooling rate in nucleate boiling decreases with the decreasing surface temperature. The lower temperature boundary of the nucleate boiling is determined by the minimum wall superheat (Δ*T* = 18°C) required to maintain vapor bubble nucleation and growth within the impinging droplets. Finally (after about 290 s), the film evaporation or single-phase forced convection will exist below the boundary where the heat flux is about 45 W/cm^2^ at *G* = 8.92 × 10^−4^ kg/cm^2^s.

#### Nanofluids (at a definite mass flux)

**Figure 5:**
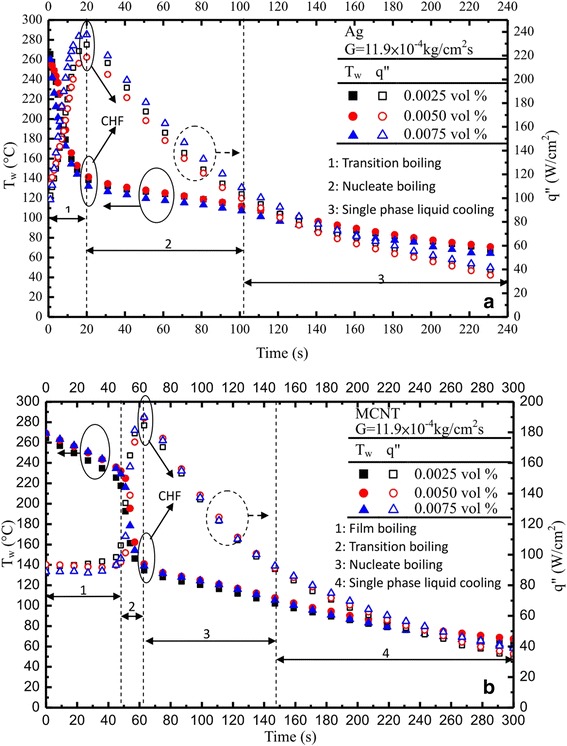
**Cooling and boiling curves: (a) nanofluids (Ag), (b) nanofluids (MCNT).**

Effect of nanofluids at *G* = 11.9 × 10^−4^ kg/cm^2^s can be seen in Figure 5a,b for nanofluid (Ag) and nanofluid (MCNT) with different volume fractions of 0.0025 to 0.0075 vol.%, respectively. The cooling history for both surface temperature and heat flux was recorded. Generally, they have an increase (decrease) in heat flux (surface temperature) as the nanoparticle volume fraction increases for both Ag/MCNT nanofluids except *ϕ* = 0.0050% for Ag nanofluids. Such an inconsistency may be due to transient instability or experimental errors. Surprisingly, it was found that Ag and MCNT nanofluids significantly increased the heat transfer performance in spray cooling in comparison with DI water, as shown in Figure 4. The corresponding boiling curves for Ag nanofluids did not exhibit a traditional film boiling regime. This was possibly caused by the much higher heat transport of Ag nanofluids due to a much more uniform dispersed solution of the Ag nanofluid as compared to that of MCNT nanofluids also evidenced by Figure 1a,d. Moreover, when the mass flow rate of the Ag nanofluids, supplied to the interface through the porous medium due to quick accumulation of Ag nanoparticles during the evaporation processes, was greater than the mass flow rate of the vapor leaving the interface, a stable vapor film was no longer formed on the heated surface. Consequently, a film boiling mode does not occur and nucleate boiling starts immediately after the spray is impinged upon the surface. Meanwhile, for MCNT nanofluids, the opposite trend would occur, which results in the vapor film thickness increasing. In summary, the characteristics of a nanofluid depend on a number of parameters, including the following: the properties of the base fluid and the dispersed phase, nanoparticle size (Ag/MCNT, this study), concentration, and morphology. These would result in the boiling curves of the Ag nanofluids to be unlike DI water and MCNT nanofluids; for example, there was no distinct film boiling regime and a much shorter elapsed/or cooling time. Upon close examination, although it was found that MCNT nanofluids have a little higher thermal conductivity increase than Ag nanofluids, which can be found later, Ag nanofluids have a superior cooling performance to that of MCNT nanofluids. This will be assessed later.

**Figure 6:**
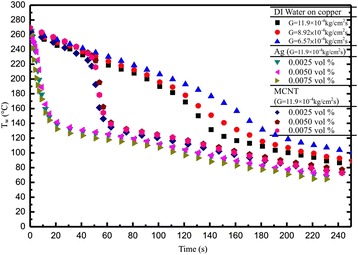
**Cooling curves for the present three fluids used.**

Moreover, as the nanofluids were sprayed, Ag/or MCNT nanoparticles began to stick to the heated surface. Subsequent impinging spray droplets dispersed some of these nanoparticles from the point of direct impact on the surface but not completely off the heated surface. Therefore, a circular band of nanoparticles was observed sticking around the regime of direct impact of the spray cone on the heated surface. Based on the above statements, the cooling rate of each of the three volume concentrations of nanofluids differed. The data strongly indicated dependence of the cooling performance of the nanofluids on the nanoparticle volume concentration. If nanoparticles can be prevented from sticking to the heated surfaces, enhanced cooling performance could be achieved with nanofluids. Figure 6 shows a summary of the results for cooling curves for the base fluid (DI water) for three different mass flux MCNT nanofluids (*G* = 11.9 × 10^−4^ kg/cm^2^s) and Ag nanofluids (*G* = 11.9 × 10^−4^ kg/cm^2^s) altogether in the same plot. Although the effect of the nanoparticle volume fraction is limited, differences among them can be clearly noted again. The effect of nanofluids on cooling curves is quite significant as well, especially when the cooling time is less than 120 s.

Furthermore, the higher nanoparticle concentration would cause a higher cooling performance for both nanofluids due to the effective thermal conductivity and thermal diffusivity increase with particle concentration. In addition, based on the flow visualization, the thermal boundary layer thickness may thus deteriorate due to nanoparticle migration which resulted in the nano-uniform distribution of viscosity and viscosity changes to intensify the turbulence. With an increase in the volume fraction of the nanofluids from 0.0025% to 0.0050%, the cooling performance increases significantly; however, at a nanoparticle concentration of 0.0075 vol.%, due to the possible deposition of the nanoparticles on the surface, lower heat transfer performance was expected. This was observed by measuring the roughness of the copper surface after experiments.

### Boiling curves (steady state)

**Figure 7:**
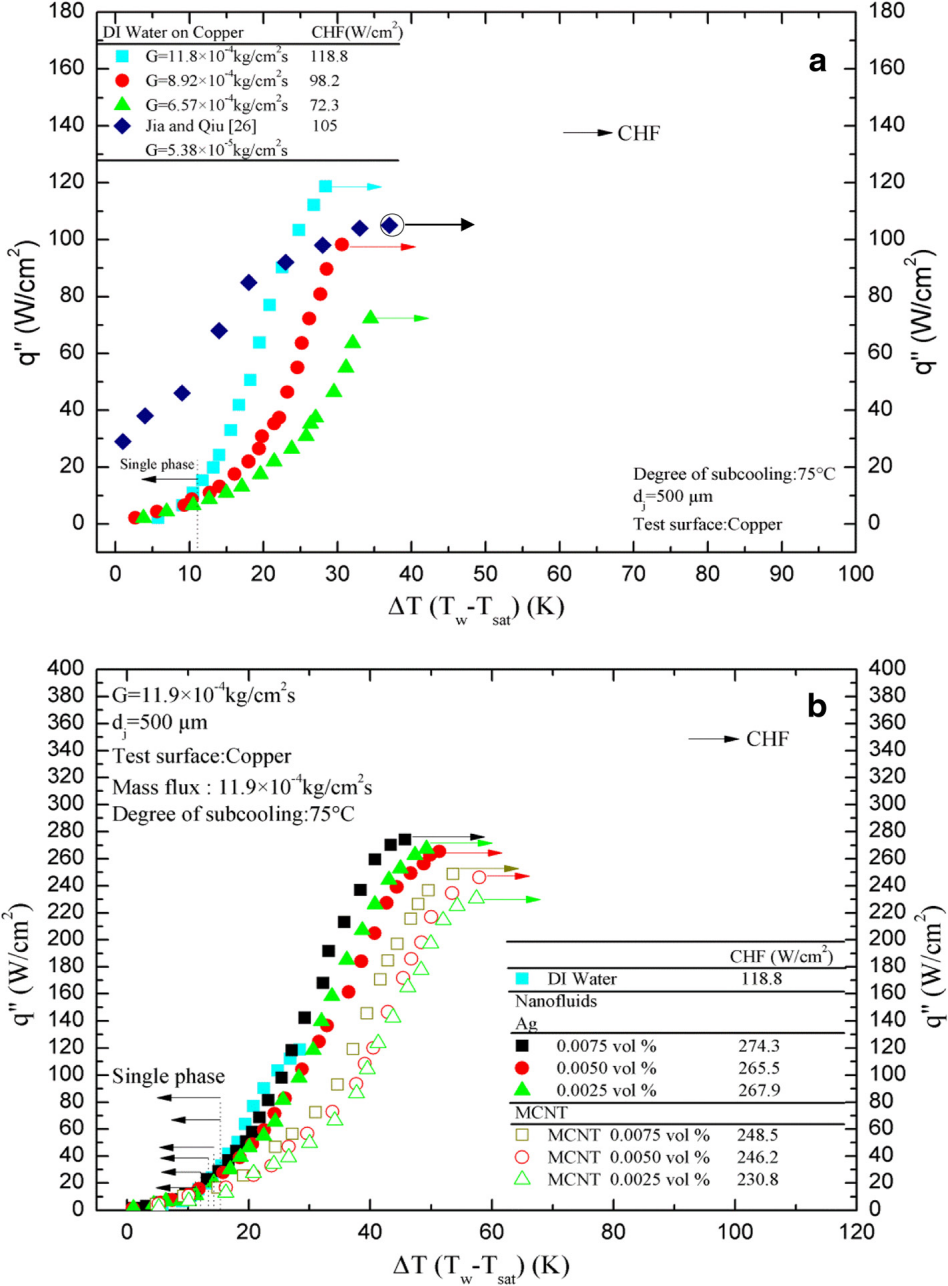
**Steady state boiling curve: (a) DI water, (b) nanofluids (Ag/MCNT).**

DI water at 25°C was sprayed onto a heated surface at varying pressures and flow rates. The heat flux and wall superheat were determined simultaneously as the wall surface was gradually heated. Figure 7a shows the heat transfer characteristics for *G* = 6.57 × 10^−4^, 8.92 × 10^−4^, and 11.9 × 10^−4^ kg/cm^2^s. There seems to be two distinct regions in the curves shown in Figure 7a, as also reported by Hsieh et al. [23]. In the first region, single-phase forced convection and evaporation are the modes of heat transfer. As the heat flux increased gradually, the slope of the curve changed, at which point nucleate boiling began. The heat flux kept increasing until the CHF was observed. From Figure 7a, drawn between wall superheat and the heat flux, it is observed that the boiling curves for DI water shifts towards the left, indicating that the wall superheat decreases with increasing mass flux, with a corresponding CHF increase; 118.8 W/cm^2^ at *G* = 11.9 × 10^−4^ kg/cm^2^s, 98.2 W/cm^2^ at *G* = 8.92 × 10^−4^ kg/cm^2^s, and 72.3 W/cm^2^ at *G* = 6.57 × 10^−4^ kg/cm^2^s, respectively.

Similar results were obtained, except that the boiling heat transfer of both nanofluids is expected to be higher than that of DI water, as depicted in Figure 7b, which is not mainly due to the fact that the thermal conductivity of the Ag and MCNT nanofluid was greater than that of the base fluid (DI water) as in most previous studies. On the contrary, the possible enhancement mechanism was caused by the microscale mixing due to the local turbulence induced by the nanoparticles rolling and the tumbling of Ag/MCNT nanoparticles or tube agglomeration [24]. Figure 7b shows that at *G* = 11.9 × 10^−4^ kg/cm^2^s, Ag nanofluids have a superior heat transfer performance compared to that of MCNT nanofluids. Furthermore, for a given heat flux, the wall superheat decreases with the increasing volume fraction of either Ag or MCNT, respectively. It was also observed that the CHF of nanofluids increases with the increase of Ag/MCNT concentrations; this result differs from those of Kathiravan et al. [25] who reported a contrary trend. This is perhaps because our volume fraction of nanofluids is much smaller than theirs, where the nanoparticle deposition impedes the heat transfer. The present CHF found is up to 248.5 W/cm^2^ and 274.3 W/cm^2^ for 0.0075 vol.% of MCNT and Ag, respectively, at *G* = 11.9 × 10^−4^ kg/cm^2^s. Consequently, the value of CHF for nanofluids can be up to 2.0 to 2.4 times greater than that of pure DI water. We found that the boiling-induced deposition of the nanoparticles changed the heater surface to have wettability for effectively extending the CHF. Also shown in Figure 7a is pure DI water data from Jia and Qiu [26] with a lower mass flux for comparison. The present results are obviously superior to that of Jia and Qiu [26] from the viewpoints of the onset of nucleate boiling (ONB) and CHF due to a higher mass flux.

Overall, from Figures 4, 5, 6, and 7, one may find that there is a little bit of difference in the values of ONB between the steady state (approximately 14°C) and transient (approximately 7°C) results. These differences (≤6.6%) are within the measurement uncertainty. However, differences in CHF seem significant. Possible reasons for both are due to violent processes like vigorous vapor generation, where the nanoparticles are detached (are not deposited on anymore) from the heated surface causing the number of microsized cavities to increase, which then decreases the wall superheat, and perhaps due to the nanofluid particle deposits, are not ready to settle, respectively, during the transient time.

### Heat transfer coefficient (*h*) and cooling enhancement

**Figure 8:**
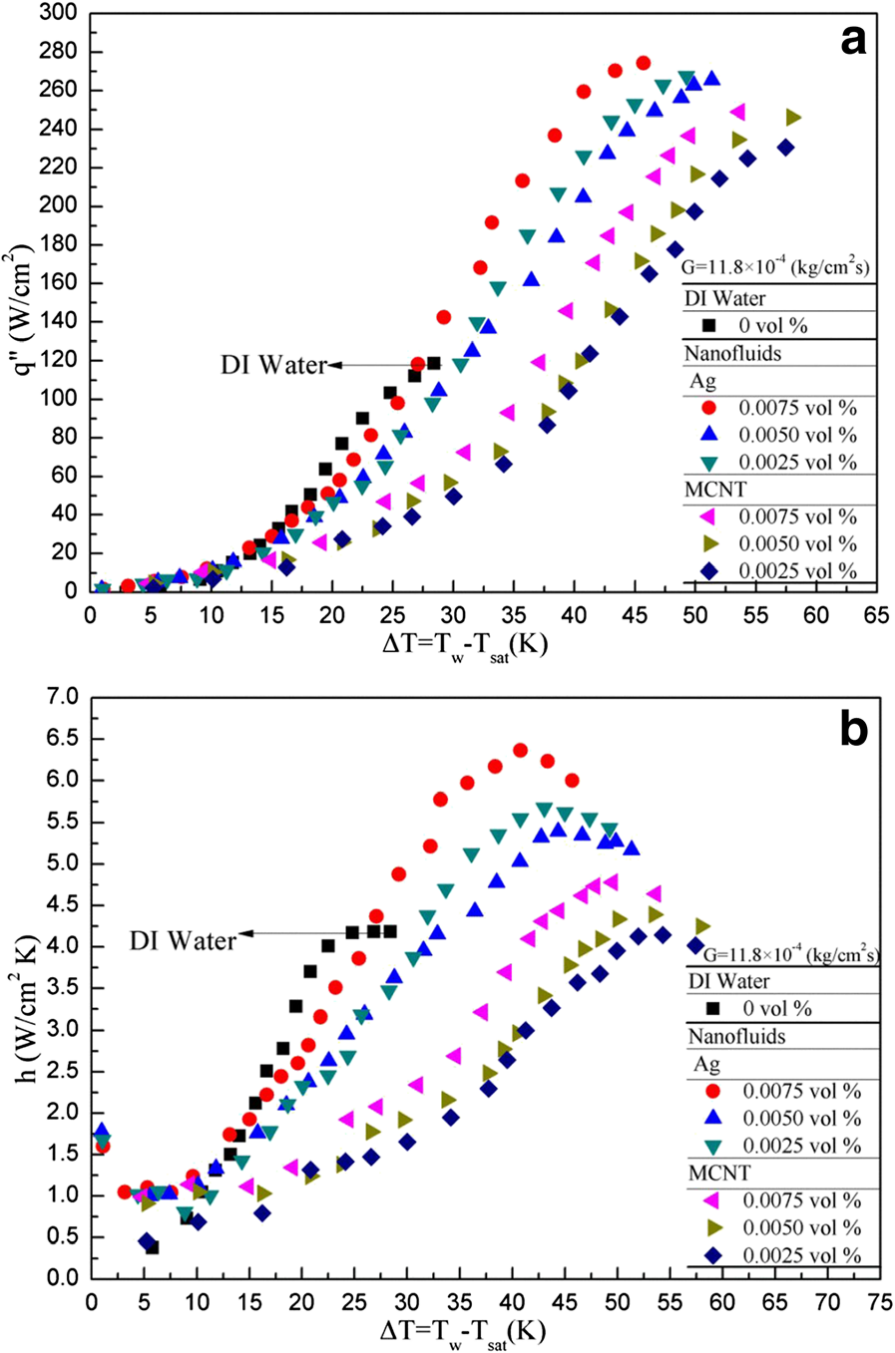
**Nanofluids vs DI water: (a)** ***q*** **-Δ** ***T*** **, (b)** ***h*** **-Δ** ***T*** **.**

**Figure 9:**
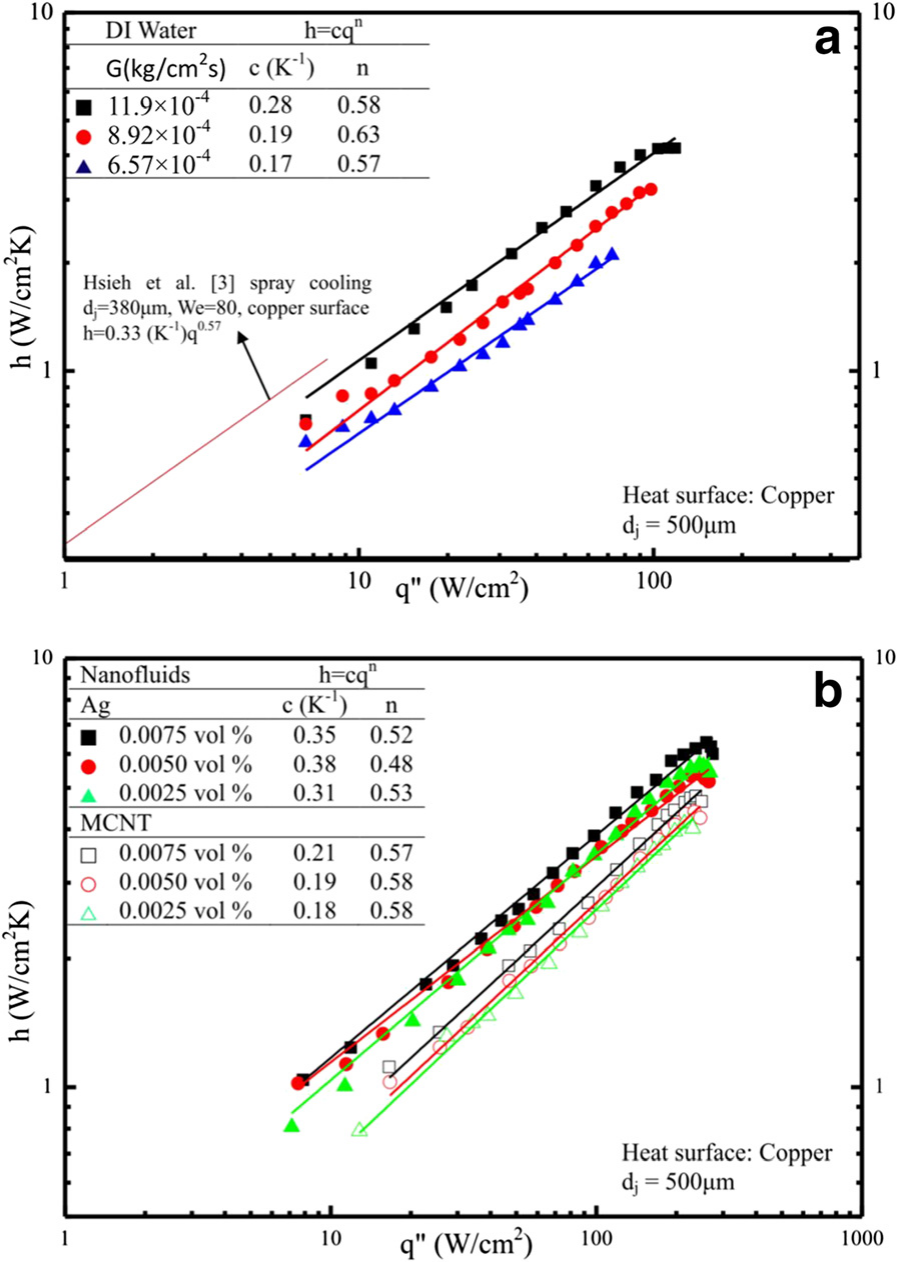
***h*** - ***q*** **″ curves: (a) DI water, (b) nanofluids (Ag/MCNT).**

**Figure 10:**
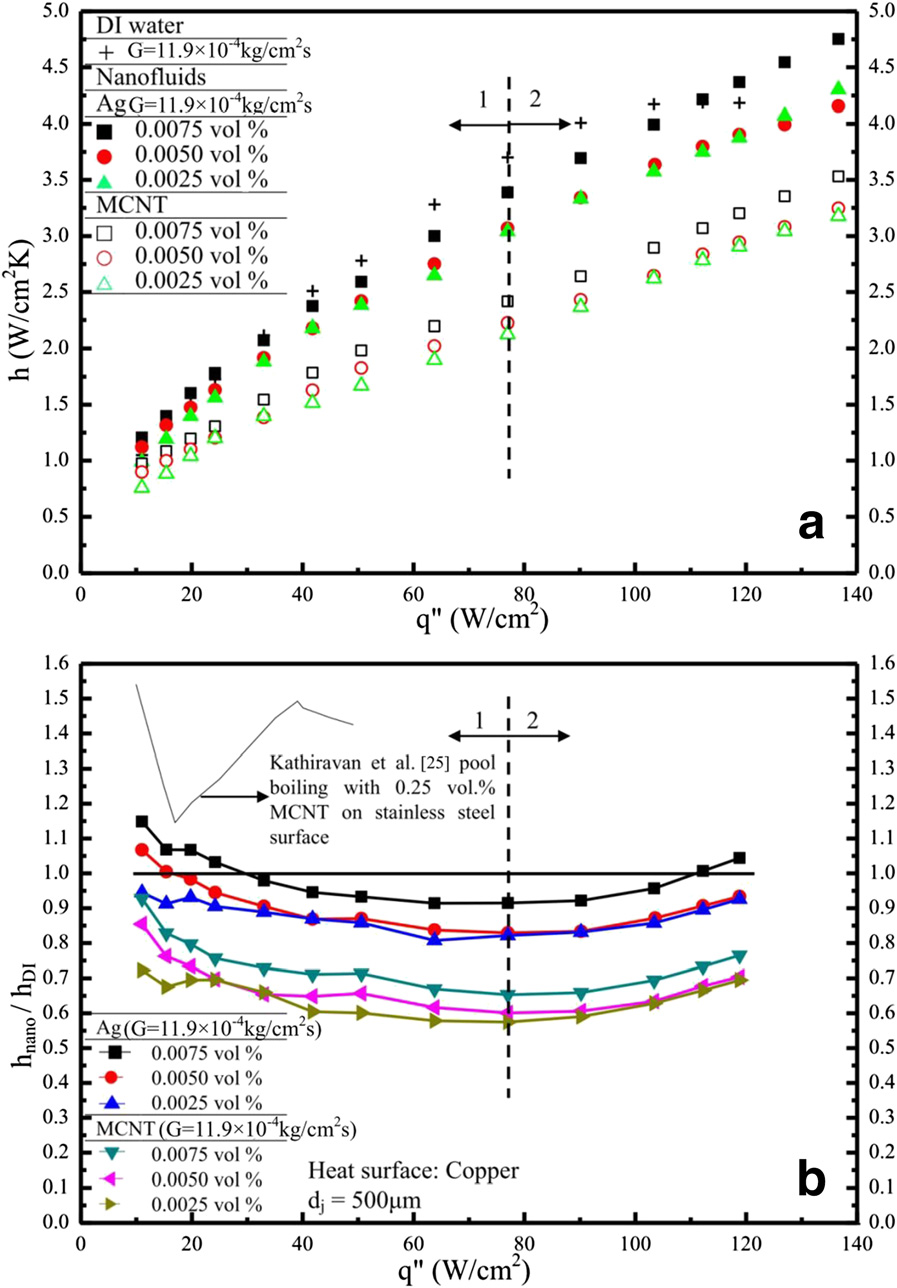
**DI water and nanofluids: (a)** ***h*** **vs** ***q*** **″, (b)** ***h***_nano_ **(nanofluid)**/***h***_DI_ **(DI water) vs** ***q*** **″.**

Figure 8 presents a comparison on heat transfer of the present nanofluids with that of DI water. Surprisingly, the heat transfer enhancement is not clearly noted at the same mass flux, which means that the nanofluids with different volume fraction under study is not superior to that of DI water. This result quite coincides with some of previous studies [12-14]. However, the trend seems opposite due to the following reasons: there are two possible major mechanisms to involve nanofluid heat transfer during spray cooling. (i) Fluid mechanics; high volume fraction nanofluid has a higher duration time when it impacts upon the heater surface which deteriorate the heat transfer; however, (ii) the thermal conductivity of the nanofluid, most likely, increases with the increased volume fraction of the nanofluid for this study. The net heat transfer effect is based on the counter balance of the above two factors. Due to the different nanofluids as well as different concentration and different operating conditions (e.g. temperature, etc.) from the previous published papers [12-14]), it seems that they may come out different conclusions. Figure 9 displays the *h* vs. *q*″ distribution for DI water with different Weber numbers and for nanofluids of Ag and MCNT with different volume fractions. Note that the *h* here indicates a local value (function of heat flux). This *h* increases as the heat flux increases in the power (exponent) of nearly 0.59 ± 0.04 for DI water, as shown in Figure 9a. This value is quite similar to that in Hsieh et al. [3] also included in Figure 9a. Similarly, the same *h* vs *q*″ trend is obtained as illustrated in Figure 9b with a slightly smaller exponent (0.55) found for Ag and MCNT nanofluids. However, the heat transfer coefficient value *h* is generally much higher than that of DI water and is about 4.3 W/cm^2^K for Ag nanofluids at *q*″ = 100 W/cm^2^ and *G* = 11.9 × 10^−4^ kg/cm^2^s, for example. On the other hand, *h* (at the same *q* and *G*) is about 3.8 W/cm^2^K for DI water. A more detailed *h* and comparison of (*h*_nano_**/***h*_DI_) as a function of heat flux was plotted and shown in Figure 10a,b, respectively. It was found that there seems to be no significant difference in the heat transfer coefficient between DI water and MCNT nanofluid. In fact, in Figure 10b, for a given heat flux, *h*_nano_ is lower than *h*_DI_ in most cases. But, nevertheless, it can also be seen that two different trends (one is a positive and one is a negative slope) are found for the dependence of *h* on *q*″. From Figure 10a,b, one may conclude that the application of nanofluids’ cooling is very suitable for high power devices because *h* increases as *q* increases when the cooling demand of the power devices exceeds about 80 W/cm^2^. Furthermore, the effect of nanofluids with different volume fractions can be seen, although some fluctuations with an average value of 1.1 to 1.2 for MCNT and Ag nanofluids, respectively, were also observed, as illustrated in Figure 10b.

**Figure 11:**
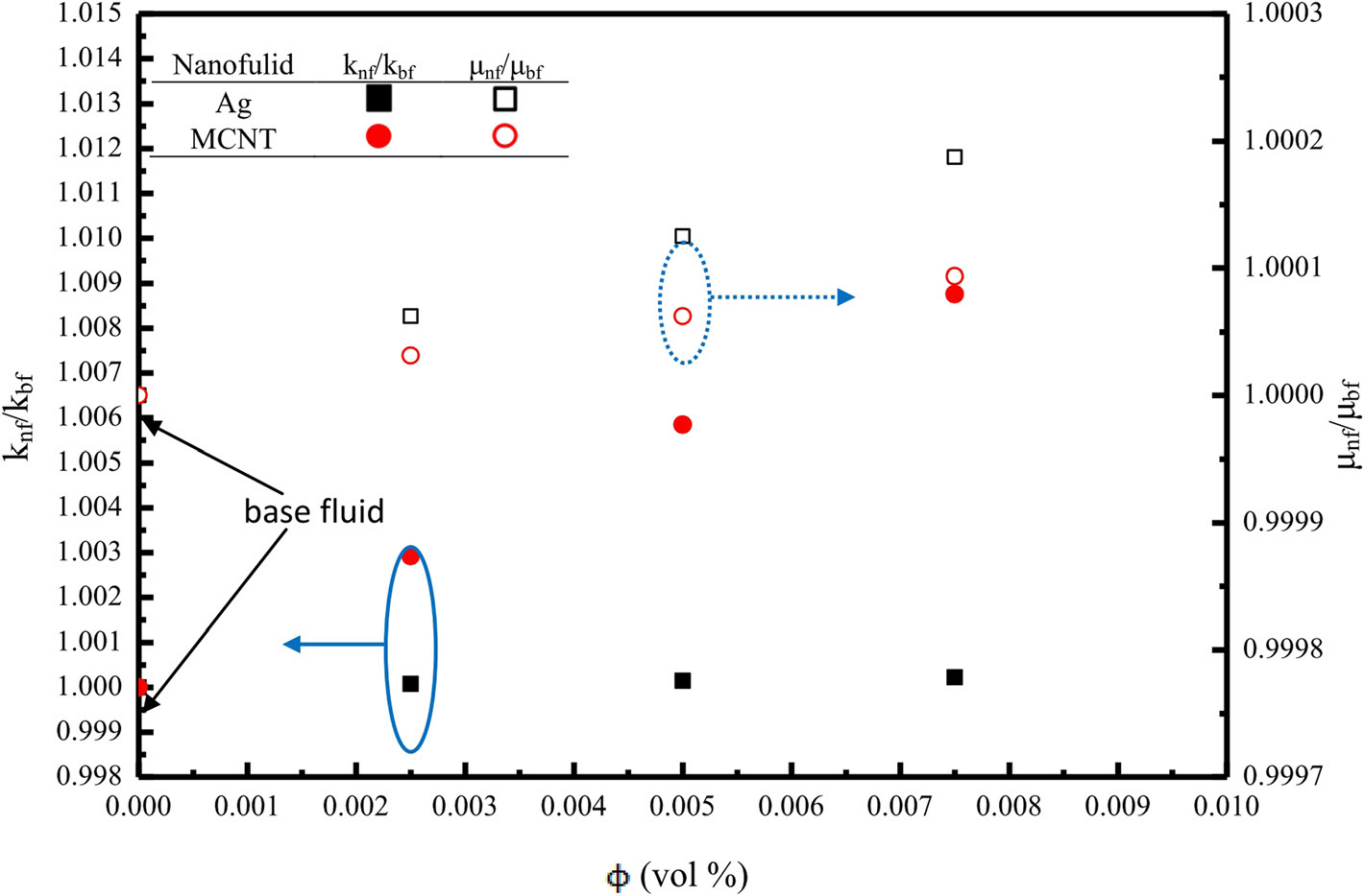
**Relative thermal conductivity and viscosity of nanofluids vs function of nanoparticles volume fraction (** ***ϕ*** **).**

**Figure 12:**
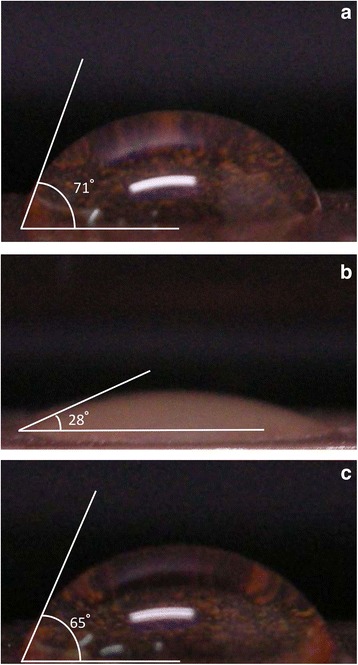
**The contact angle of heated surface for different working medium: (a) DI water, (b) nanofluids (Ag 0.0075 vol.%), (c) nanofluids (MCNT 0.0075 vol.%).**

Although it is clearly seen in Figure 11 that the thermal conductivity of nanofluids of MCNT is higher than that of Ag nanofluids, the surface area of Ag nanofluids is greater than that of MCNT nanofluids (see Table 3). Moreover, the enhancement of the relative thermal conductivity for both Ag/MCNT nanofluids seems low due to the present low volume fraction. This may explain why the cooling/heat transfer performance displayed by Ag nanofluids is superior to that of MCNT nanofluids. Figure 11 also shows the calculated effective viscosity variation (from Table 3) as a function of the volume fraction of Ag and MCNT nanofluids. The trend is opposite to that of the calculated thermal conductivity. In addition, the viscosity changes for Ag nanofluids are greater than those for MCNT nanofluids. Therefore, as stated previously, this would thicken/thin the boundary layer and, therefore, enhance the turbulence, resulting in better heat transfer. The present results are most likely based on speculation rather than scientific evidence. Nanofluids with much lower particle concentrations were found to have a better heat transfer. This is partly because when evaporation occurred, the surface condition was changed with nanoparticles left on the surface and partly because a longer spray duration time on the surface with low particle concentration. For higher particle concentration, the particles stuck on the surface and the nanoparticles with a relatively long duration time would deteriorate the heat transfer. This may explain some inconsistency results of the previous studies [9,15]. Besides, from Table 3, the Prandtl number of Ag nanofluids is also obviously greater than that of MCNT nanofluids, which also strongly indicates that more bubbles (visualized) are generated in Ag nanofluids than those in MCNT nanofluids due to the fact that Ag enhances boiling heat transfer more significantly (e.g. more active nucleation sites, low superheats, etc.) than those of MCNT nanofluids due to wettability and the surface tension of the Ag nanofluids, as assessed by the smaller contact angle (28° for Ag nanofluids), as shown in Figure 12.

### Critical heat flux in spray cooling of nanofluids

It is known that the CHF is highly dependent on mass flux or the Weber number, spray characteristics, and surface conditions. The present study was focused on the former two factors but with a lower mass flux.

**Figure 13:**
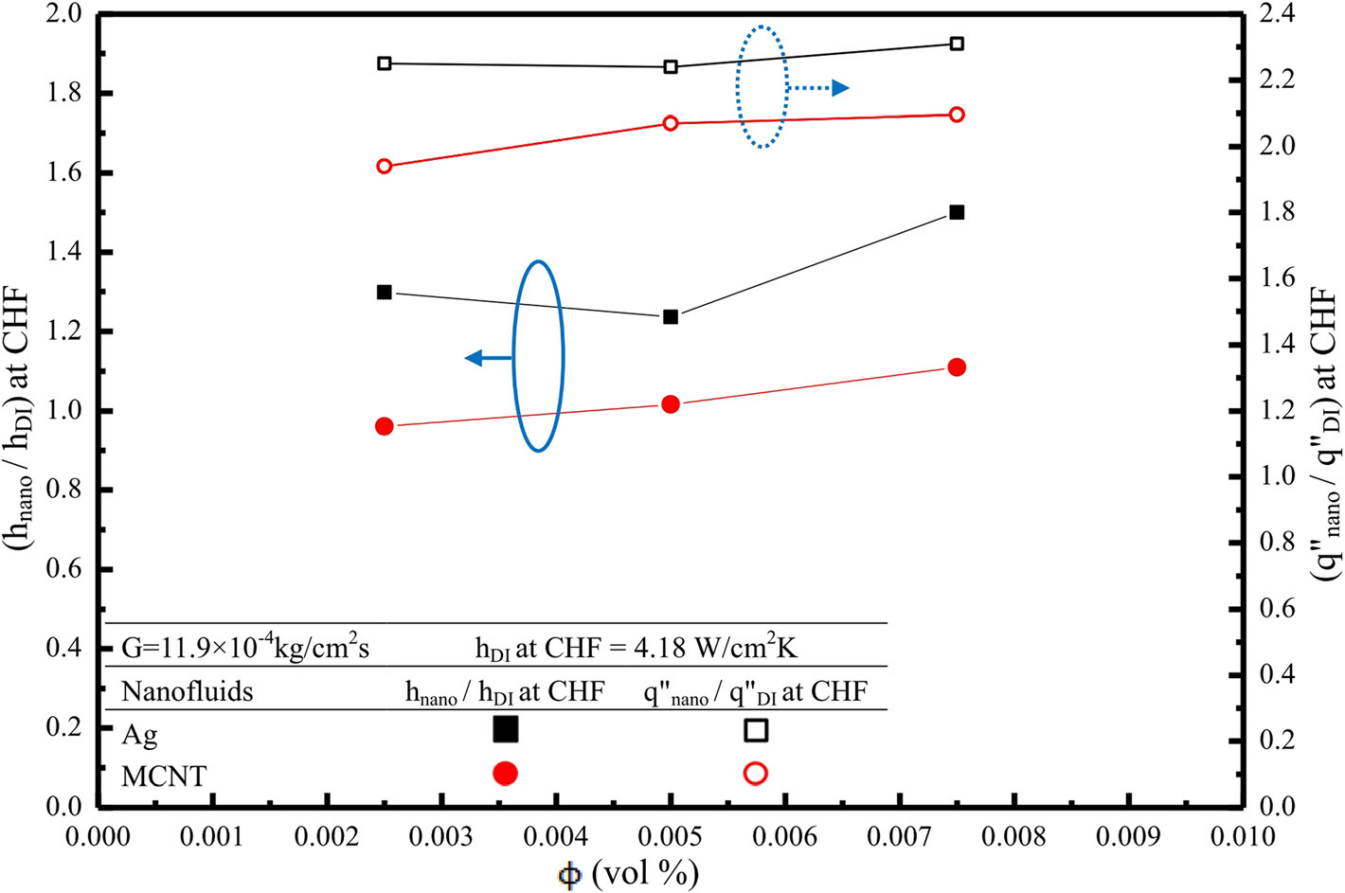
***h***_nano_ **/** ***h***_DI_ **(** ***q***″**nano/** ***q***″**DI) at CHF vs volume fraction (** ***ϕ*** **).**

With differing tendencies in spray cooling curves for nanofluids, the value of the critical heat flux (CHF) is enhanced in nanofluids with different nanoparticle volume fractions (*ϕ*), following Figure 13. For Ag nanofluids, *ϕ* = 0.0025% would give a 130% increase in CHF; however, increasing nanofluid concentration three times, *ϕ* = 0.0075% does not increase CHF with the same trend due to complex (nonlinear) physical phenomena, as expected. The results are encouraging, showing a 200% to 250% rise in CHF over spray cooling of pure DI water for both Ag and MCNT nanofluids. An increase in bubble departure diameter was also observed (not shown). This may be caused by the effective surface tension that the present nanofluids have. This finding is quite similar to those of previous investigations [12,25]. The surface deposition of nanoparticles affects surface characteristics. It is suggested that surface wettability and morphology are key parameters as to the CHF enhancement. If the surface is highly wettable, the surface dry out may be delayed which indicates that the CHF increases. Actually, in the present study, the effect of nanoparticles on CHF is clearly noted because the nanoparticle deposit on the plate surface may act as a conductivity layer affecting the CHF for nanofluids; the effect on CHF values is different for Ag and MCNT nanoparticles. In fact, due to a much smaller contact angle for Ag nanofluids (highly wettable) compared to that of MCNT nanofluids (28° vs 65°) (see Figure 12), the CHF value of Ag nanofluids would be higher, which is also attested by Gerardi et al. [27]. On the other hand, heat transfer enhancement of nanoparticles on *h* cannot be clearly seen, as shown in Figure 6b; there is only a 5% to 15% increase for Ag nanofluids for some *q″* while the CHF does not. It may be concluded that the CHF plays an important role as far as enhancement of cooling performance with nanofluid spray cooling is concerned.

In addition to the above-stated parameters and variables under study, based on a review work [28], the nanoparticle size may also have impact on the nanofluid behavior because it would affect the thermophysical properties of the nanofluids. Since the present study has mainly focused on the effect of the volume fraction of the nanofluids especially for very low volume fraction like *ϕ* = 0.0025%, additional experiments in this issue could not be done at this stage. Future and further study may include this regard.

## Conclusions

Spray cooling characteristics of nanofluids of Ag and MCNT nanoparticles of very low volume fractions (*ϕ*) of 0.0025%, 0.0050%, and 0.0075% were extensively studied for DI water with a constant nozzle-to-surface distance of 25 mm via a 0.5-mm micronozzle. Both steady and transient experiments were carried out; and relevant data for the two-phase heat transfer coefficient, boiling curve**/**cooling history, as well as the corresponding CHF were presented with a low mass flow flux 11.9 × 10^−4^ kg/cm^2^s; and the effect of nanofluids on these parameters was discussed. The salient features are as follows:

1. The effect of nanofluids, especially for very low concentrations (≤0.0075 vol.%), was clearly noted with different cooling regimes displayed. The major heat transfer enhancement mechanism can be attributed to the increased mixing rather than to the traditional higher thermal conductivity of the nanofluid.
2. Heat transfer increase by the type of the nanofluids used was not clearly found; only at low heat flux (≤25 W/cm^2^) and high heat flux (≥115 W/cm^2^) that the heat transfer enhancement occurs for Ag nanofluids with 0.0075% volume fraction. However, for critical heat flux, the nanofluid enhancement was assessed as previous studies did. A correlation of *h* vs *q*″ was also developed and found *q*″ or *h* increases by adding Ag or MCNT nanoparticles. Heat transfer enhancement ratio of (*h*_nano_/*h*_DI_) was found to be strongly dependent on surface heat flux (i.e., target surface temperature) with the lowest value occurred at *q*″ = 80 W/cm^2^ for each case under study.
3. The effect of nanofluids on CHF values is quite significant. Heat transfer enhancement can be up to 2.4 times that of the base fluid as far as CHF values are concerned.
4. It is surprising that the Ag nanofluids show superior heat transfer compared to that of MCNT nanofluids in a two-phase nucleate boiling regime, although the effective thermal conductivity of the latter is higher than that of the former. Most possibly, it is because the Ag nanofluid solution has an evenly dispersed phase without any agglomeration, compared to that of MCNT nanofluids.
5. The present CHF was found to be 248.5 W/cm^2^ and 274.3 W/cm^2^ for 0.0075 vol.% of MCNT and Ag nanofluids, respectively, at *G* = 11.9 × 10^−4^ kg/cm^2^s.
6. The heat transfer coefficient (*h*) increases as the spray mass flux increases, as expected.

## Nomenclature

- *Cp*: specific heat, kJ/kg K
- CHF: critical heat flux, W/cm^2^
- *d*: the diameter of MCNT, nm
- *d*_32_: Sauter mean diameter, ${\displaystyle \sum {d}_i^3}/{\displaystyle \sum {d}_i^2}$, m
- *G*: mass flux, kg/cm^2^s
- *h*: heat transfer coefficient, W/m^2^K
- $\overline{h}$: average heat transfer coefficient, W/m^2^K
- *k*: thermal conductivity, W/mK
- *l*: the length of MCNT, nm
- *k*_cu_: thermal conductivity of the copper plate, W/mK
- *q*″: heat flux, W/cm^2^
- *x*, *y*, *z*: coordinates, m
- *T*: temperature, K
- *u*_*o*_: impact velocity, m/s
- *u*_*j*_: spray velocity at nozzle exit, m/s
- We: Weber number, ρu_0_^2^d_32_/σ

### Greek symbols

- *ρ*: density of liquid, kg/m^3^
- *σ*: surface tension, N/m
- *μ*: viscosity of liquid, Ns/m^2^
- *ϕ*: volume fraction of nanoparticle
- Δ*P*: pressure drop across the nozzle, N/m^2^

### Subscript

- c: spray liquid layer
- cu: copper
- j: nozzle exit
- w: target surface
- o: impact
- sat: saturation
- nf: nanofluid
- bf: basefluid
- np: nanoparticle

## Abbreviations

CHF: critical heat flux
MCNT: multi-walled carbon nanotube
ONB: onset of nucleate boiling
